# Editorial: Puzzle pieces from malaria vaccine clinical trials

**DOI:** 10.3389/fimmu.2023.1138763

**Published:** 2023-01-30

**Authors:** Nirianne Marie Q. Palacpac, Alfred B. Tiono, Benjamin Mordmüller, Takafumi Tsuboi

**Affiliations:** ^1^ Department of Malaria Vaccine Development, Research Institute for Microbial Diseases, Osaka University, Suita, Osaka, Japan; ^2^ Groupe de Recherche Action en Santé (GRAS), Ouagadougou, Burkina Faso; ^3^ Department of Medical Microbiology, Radboud University Medical Center, Nijmegen, Netherlands; ^4^ Division of Cell-Free Sciences, Proteo-Science Center, Ehime University, Matsuyama, Japan

**Keywords:** malaria, *Plasmodium*, vaccine, controlled human malaria infection, clinical trials

The recent endorsement of RTS,S/AS01 for broad use in children residing in regions with moderate to high malaria transmission is a key milestone in the fight against malaria (1, 2). However, there is still a lot more work to be done. Clinical trials can be challenging in terms of study design, ethics, costs, and field implementation (logistics, recruitment of human volunteers and retention, societal perceptions, etc.), but are critical for evidence generation, new tools and innovative technological approaches. Careful analysis and insight make each clinical trial an important puzzle piece providing clues to help us understand limitations/knowledge gaps that are stumbling blocks to safe, stable, easy-to-administer, cost-effective vaccines. This *Research Topic* also highlights an important key-ingredient of successful research: the extensive collaborations and multidisciplinary/transdisciplinary consortiums at the root of each activity.

## The *Plasmodium falciparum* life cycle: peaks and troughs of vaccine development

The complexity of the parasite lifecycle; gaps in our understanding on the interactions of the parasite and host; and the amazing capacity of malaria-infected red blood cell for immune subversion and immunosuppression (3) are high hurdles for vaccine development. Ramjith et al., 2022 developed mathematical models, presented power analysis considerations, and made an online tool to allow data analysis and sample size estimation when conducting trials for transmission-blocking interventions. The authors describe what data are needed for either an assessment of transmission-blocking activity or transmission-reducing activity and where power can be increased while considering the many confounders involved. The models seek to maximize the informativeness of future transmission-blocking intervention trials, and allow pre- and post-intervention comparisons.

Nunes-Cabaço et al., 2022 traced the history of the clinical assessment of whole-sporozoite malaria vaccines from its earliest concept in 1967. Several milestones were discussed, including the achievements in PfSPZ (*Pf* sporozoite) vaccine production and controlled human malaria infection (CHMI) studies. To date, PfSPZ vaccines seem highly protective in malaria-naïve adults but somewhat less active in African adults. First results from studies in infants and small children have been disappointing.

Sex has recently gathered attention as a variable that can influence immune response, vaccine efficacy and safety. Clinical trials of PfSPZ-based vaccines in the US, Germany, Kenya, Tanzania, Mali, Burkina Faso, and Equatorial Guinea showed that participants older than 11 years of age had sex-associated differences in vaccine-induced antibody response but no sex-related differences in protection (in CHMI or field clinical trials) (KC et al., 2022). Several trials also show that antibody levels against sporozoites were not predicting protection per se, and that prior malaria exposure significantly resulted in lower antibody responses, even in females with higher antibody levels than their male counterparts.

The perspective article by Owalla et al., 2022 emphasizes the need for highly sensitive parasite diagnostics in endemic settings. Indeed, low-density infections in malaria-endemic areas are common, often ignored and their influence in trial outcomes and end-point assessments remain unclear. The authors compared the current tools for determining infection status and suggest frequent dried blood spot sampling with pooled qRT-PCR as a cost-effective strategy to circumvent infection monitoring blind-spots in clinical trials and epidemiological studies.

The study by Xu et al., 2022 provides preliminary data for delivering a multi-antigen vaccine in a single vaccine formulation in the form of a multi-layer nanoparticle. The authors tested trimethyl chitosan-based layer-by-layer nano-assembly vaccine platform as a delivery vehicle for three antigens: CSP, AMA1, and MSP1. Biophysical characteristics of the delivery platform showed promise.

At the preclinical stage is another vaccine antigen, a fragment of *P. falciparum* Rh5-interacting protein (PfRipr5) (Takashima et al., 2022). PfRipr5 has been identified as a promising blood-stage vaccine candidate and is proceeding into clinical testing. The GMP-compliant recombinant protein was produced using the insect cells-baculovirus expression vector system and tested in pre-clinical model. Three human-acceptable adjuvant formulations tested head-to-head: Alhydrogel^®^, GLA-SE or CAF^®^01 showed comparable levels of anti-PfRipr5 antibodies. The highest functional activity by growth inhibition assay (GIA) was obtained in PfRipr5 with CAF^®^01.

Bougouma et al., 2022 reported the results of a phase Ib trial of the BK-SE36 vaccine candidate based on the serine repeat antigen-5 in 12- to 60-month-old children living in a malaria endemic area in Burkina Faso. The safety and immunogenicity of BK-SE36 were demonstrated in this age group for the first time. In general, the vaccine was safe and similarly immunogenic when given subcutaneously and intramuscularly; and as expected, subcutaneous vaccination led to more adverse events than the intramuscular route. The increase in IgG titers after vaccination was more pronounced in 12–24 months than in 25–60 month-old children, and a delayed third dose significantly boosted the immune response.

Looking for clues in a follow-up study after a Phase 2b multi-center clinical trial of the GMZ2 vaccine (4), in-depth anti-GMZ2 antibody responses were investigated in one of the sites where the highest incidence of malaria was observed (Dassah et al., 2022). The study showed the importance of naturally acquired immunity; the influence of age and parasite threshold at which fever is triggered; and the relatively high pre-existing anti-merozoite antibodies in Burkinabe children.

## Needles in a haystack: CHMI challenges and clues

CHMI is increasingly becoming an important tool for the clinical evaluation of candidate drugs and vaccines as well as a model to dissect the heterogeneity in immune response to malaria (5). The study by de Jong et al., 2022 assessed antibody responses in two CHMI trials (with or without *P. falciparum* gametocyte exposure) to disentangle stage-specific signals and identify responses specific to sexual stage parasites *vs* asexual stage antibody response. The study provide insight into the humoral responses to two transmission-blocking vaccine candidates (Pfs48/45 and Pfs230) and identified new antigens that may be developed as markers for gametocyte exposure.

Salkeld et al., 2022 used CHMI in attempts to mimic the field observation of blood-stage malaria immunity acquired throughout several clinical episodes. After three homologous blood-stage CHMI, majority of the subjects did not show measurable functional anti-parasite immunity based on reduced parasite growth/multiplication rate but repeat infections did show boosting of antibody responses to MSP1 and AMA1. The work demonstrated the safety of repeated CHMI with no major differences in clinical symptoms or laboratory markers of infection across primary to tertiary challenges.

Last but not the least, while clinical trials against *P. falciparum* are making progress, the highs and lows in *P. vivax* had come in trickles (6, 7). *P. vivax* is the most dominant malaria parasite throughout Asia-Pacific and South America (with detection currently increasing in sub-Saharan Africa). The species is considered a key obstacle in malaria elimination (8) because of its unique biology and absence of a routine continuous *in vitro* cultivation method that have largely restricted research efforts to develop interventions. Roobsoong et al., 2022 present the challenges of using *P. vivax*-CHMI, particularly the stringent and safe preparation of the parasites to be used, the logistics and limitations of sporozoite- and blood-stage CHMI of *P. vivax*.

## Conclusions

We hope that by highlighting progress, challenges and limitations in malaria vaccine clinical trials, this Research Topic will be useful in creating a shared vision that a malaria-free world needs concerted and evolving action.

## Author contributions

NP, AT, BM, and TT are the four editors of the Research Topic, wrote this editorial and approved the final version.
